# Overexpression of the Auxin Receptor *AFB3* in *Arabidopsis* Results in Salt Stress Resistance and the Modulation of *NAC4* and *SZF1*

**DOI:** 10.3390/ijms21249528

**Published:** 2020-12-15

**Authors:** Fernanda Garrido-Vargas, Tamara Godoy, Ricardo Tejos, José Antonio O’Brien

**Affiliations:** 1 Departamento de Genética Molecular y Microbiología, Facultad de Ciencias Biológicas, Pontificia Universidad Católica de Chile, Santiago 8331150, Chile; fsgarrido@uc.cl; 2 Laboratorio de Biotecnología Celular, Facultad de Recursos Naturales Renovables, Universidad Arturo Prat, Iquique 1100000, Chile; tamarandrea3190@gmail.com (T.G.); rtejos@unap.cl (R.T.); 3 Departamento de Fruticultura y Enología, Facultad de Agronomía e Ingeniería Forestal, Pontificia Universidad Católica de Chile, Santiago 8331150, Chile

**Keywords:** *Arabidopsis*, Auxin, Auxin receptors, root architecture, transcription factors, salinity

## Abstract

Soil salinity is a key problem for crop production worldwide. High salt concentration in soil negatively modulates plant growth and development. In roots, salinity affects the growth and development of both primary and lateral roots. The phytohormone auxin regulates various developmental processes during the plant’s life cycle, including several aspects of root architecture. Auxin signaling involves the perception by specialized receptors which module several regulatory pathways. Despite their redundancy, previous studies have shown that their functions can also be context-specific depending on tissue, developmental or environmental cues. Here we show that the over-expression of Auxin Signaling F-Box 3 receptor results in an increased resistance to salinity in terms of root architecture and germination. We also studied possible downstream signaling components to further characterize the role of auxin in response to salt stress. We identify the transcription factor SZF1 as a key component in auxin-dependent salt stress response through the regulation of NAC4. These results give lights of an auxin-dependent mechanism that leads to the modulation of root system architecture in response to salt identifying a hormonal cascade important for stress response.

## 1. Introduction

In order to survive plants must be able to respond and adapt to different environmental changes [1]. Thus, challenging environmental conditions can be a limiting factor for plant growth and can negatively affect their development, reproduction, and in extreme cases, survival. These stimuli include extreme temperatures, light intensity, nutritional imbalance, heavy metals concentration, osmotic stress due to high or low water availability, and high salt concentration in soils [2]. In arid and semi-arid regions, drought and soil salinity are among the most detrimental problems in agriculture [3]. The impact of high salinity in the soil can be observed throughout the life cycle from seed germination to postembryonic development. At the root level, salinity induces a reprogramming of growth, thus modifying the root architecture allowing plants to circumvent salt-rich patches within the soil hence reducing potential damages [4,5]. These salt-induced morphological changes involve a concentration-dependent inhibition of primary root elongation under mild-salinity, while high salt concentrations strongly inhibit root growth and lateral root formation [6,7,8,9]. These adaptative responses are coordinated primarily by plant hormones such as abscisic acid (ABA) and auxins, among others [5,6,10,11,12].

Auxin has a key role in the modification of root architecture during adaptative responses in plants [11]. In the canonical auxin signaling pathway, the binding of auxin to their receptors activates the E3 ubiquitin ligase complex SCF, leading to degradation of AUX/IAA transcriptional repressors. This degradation allows AUXIN RESPONSE FACTORS (ARFs) to modulate the expression of auxin-responsive genes [13,14]. In *Arabidopsis thaliana*, a plant model species, the canonical auxin perception comprises six receptors that lead to the activation of target genes: TRANSPORT INHIBITOR RESPONSE 1 (TIR1) and five AUXIN F-BOX (AFB) proteins, AFB1 to AFB5 [15]. Interestingly, the accumulation and differential perception of auxins in the root has a fundamental role in the control of several types of abiotic stress, including salt stress [8,16,17,18,19].

It has been described that while *TIR1* is up-regulated after 6 h treatment with NaCl [16], at the protein level TIR1 and AFB2 are downregulated after 4 h treatment of NaCl [20]. Moreover, the mechanism regulating *TIR1/AFB2* expression in response to salt has been described to involve posttranscriptional modulation by the microRNA *miR393* [16,20]. Interestingly, the over-expression of a resistant-to-degradation form of TIR1 (mTIR1) confers a salt-tolerant phenotype, increasing parameters such as lateral root density, germination rate, and Na^+^ exclusion [16]. Although the functions of the auxin receptors are similar and may be redundant, studies of single and multiple mutants of these receptors suggest that each receptor would have stimuli-specific functions [21]. Although the contribution of TIR1 within the regulation of auxin perception has been widely studied, little is known about the role of AFB proteins in other contexts. Likewise, AFB3 has been involved in the modulation of root architecture depending on availability of nutrients such as nitrate, thus, regulating the response of several genes involved in transport and assimilation of key metabolites [22]. Under limiting nitrate conditions it has been characterized that *AFB3* has a key role and also downstream signaling components identified [23]. Accordingly, the transcription factor NAC4, a member of the NAM/ATAF/CUC (no apical meristem (NAM)/*Arabidopsis* transcription activation factor (ATAF)/cup-shaped cotyledon (CUC)) family, has been described as a signaling component downstream of AFB3 in response to nitrate [24]. Moreover, the *AFB3-NAC4* module is possibly regulated through *INDOLE-3-ACETIC ACID INDUCIBLE 14/SOLITARY ROOT* (*IAA14/SLR*) [23].

While the mechanisms involved in auxin-mediated salt response had been studied, the role of auxin receptors in response to salt stress and their potential role in the modulation of root plasticity have not been further evaluated. Moreover, the TIR1/AFBs downstream signaling components in response to salt stress are yet not well-characterized. In this work, we showed that the auxin receptor AFB3 shows a differential response to salt stress. Furthermore, we proposed that the over-expression of AFB3 would promote salt stress resistance through the regulation of downstream response components such as *NAC4* and *SZF1* in *Arabidopsis thaliana* roots.

## 2. Results

### 2.1. AFB3 Is Regulated in Response to Salt Stress in the Root Meristem

**Figure 1:**
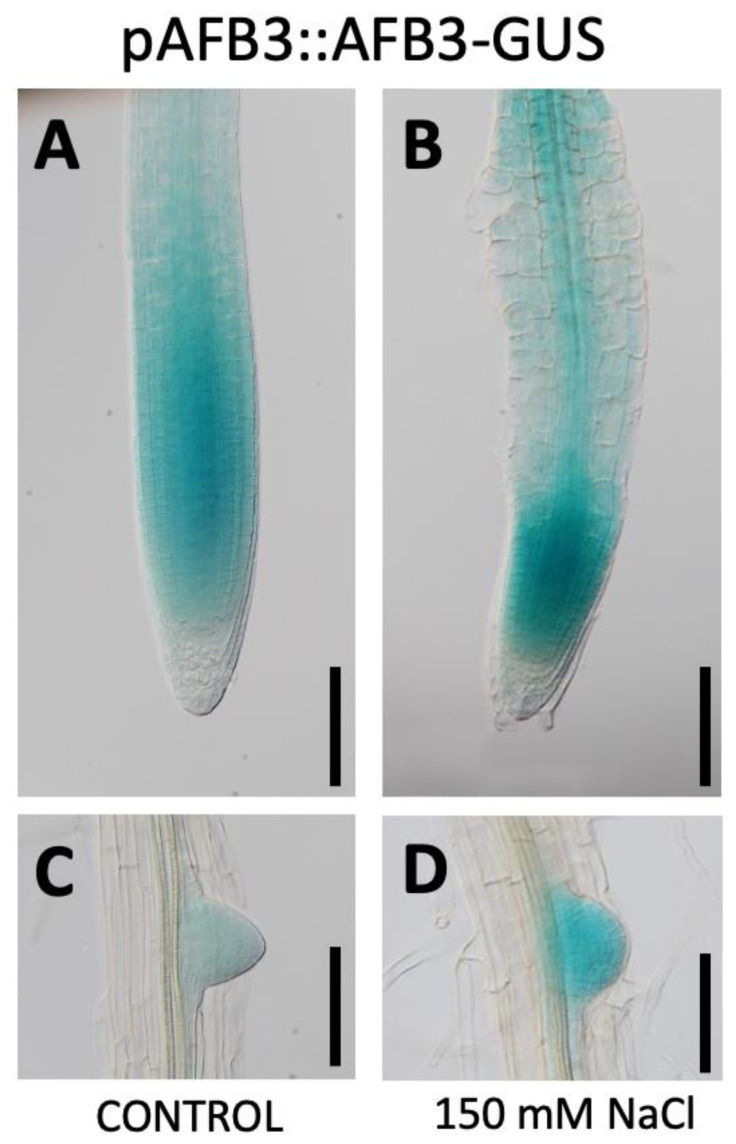
Salinity induces AFB3 protein expression in root meristem of *Arabidopsis thaliana* seedlings. Three-day-old *pAFB3::AFB3-GUS* (**A**–**D**) seedlings were transferred onto Murashige & Skoog (MS) medium supplemented with 0 (**A**,**C**) or 150 mM of NaCl (**B**,**D**) and cultivated for additional five days. At day 8 (DAS), seedlings were subjected to β-Glucuronidase (GUS) staining. A representative picture of each treatment is shown. (**A**,**B**) Meristematic/elongation zone, (**C**,**D**) Lateral root. Bar = 0.2 mm.

Preliminary data from our laboratory suggested that while TIR1 and AFB2 are downregulated at the protein level in response to salt stress [20], AFB3 might be regulated in an opposite manner. In an effort to evaluate the role of AFB3 receptor in response to salt stress, we analyzed transcriptional and post-translational *AFB3* lines fused to the *β-Glucuronidase* (*GUS*) reporter lines (*pAFB3::GUS* and *pAFB3::AFB3-GUS/afb3-4* respectively) [25]. While *AFB3* promoter is active throughout the root, and highly expressed in the columella and lateral root cap and very low in lateral roots (Supplementary Figure S1A,C), AFB3-GUS fusion protein is mainly expressed in the root apical meristem and lateral roots (Figure 1A,C) and completely absent from columella cells. In response to salt stress treatment, there is no significant up-regulation of *pAFB3::GUS* in the root meristem (Supplementary Figure S1B,D). This was further confirmed by qRT-PCR. *Arabidopsis* plants were grown for 7 days after sowing (DAS) in MS media and then transferred to MS plates (mock control) or MS plates supplemented with 150 mM NaCl. Under these experimental conditions, no significant changes in *AFB3* expression were observed in whole roots (Supplementary Figure S1E). However, when we used the *pAFB3::AFB3-GUS* reporter line, we observed a clear up-regulation of the marker in the root apical meristem and lateral root primordia (Figure 1B,D), suggesting salinity modulate AFB3 at the protein level. These results are in contrast with the regulation observed for TIR1 and AFB2, which are negatively regulated at the protein level in the root meristem under salt stress [20]. These observations suggest that AFB3 might have a key role in salt stress responses despite its mild transcriptional regulation under this stimulus. 

### 2.2. AFB3 Plays a Positive Role in Salt Stress Resistance

**Figure 2:**
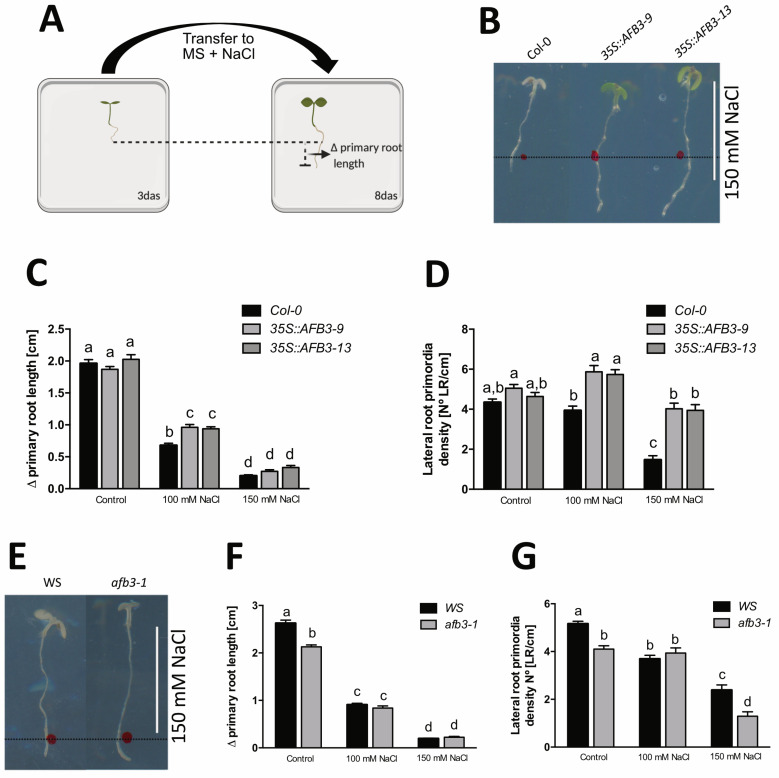
*AFB3* over-expression enhances salt stress tolerance in *Arabidopsis thaliana* roots. Three-day-old Col-0 WT, *p35S::AFB3*, WS, and *afb3-1* were transferred onto MS medium supplemented with 0, 100, or 150 mM of NaCl and cultivated for additional five days. (**A**) Schematic representation of primary root growth quantification under salt stress (Δ primary root length). (**B**) Representative Col-0 WT and *p35S::AFB3* in 150 mM NaCl are shown. (**C**,**D**) Primary root growth (**C**) and lateral root primordia density (**D**) of Col-0 WT and *p35S::AFB3* lines after transfer to NaCl-containing media were quantified. (**E**) Representative Wassilewskija (WS) and *afb3-1* seedlings in 150 mM NaCl are shown. (**F**,**G**) Primary root growth (**F**) and lateral root primordia density (**G**) of WS and *afb3-1* seedlings after transfer to NaCl-containing media were quantified. Two-way ANOVA and Bonferroni a posteriori tests were performed. a, b, c, d represents statistically significant differences with *p* < 0.05. Error bars represent the standard error of the mean. Each experiment was performed at least three independent times, with 12 seedlings each replicate. Scale: 1 cm.

*TIR1* and *AFB2* over-expression has been linked to salt stress resistance [20]. To further investigate the role of *AFB3* as a component of salt stress resistance, we generated *AFB3* overexpression *Arabidopsis* transgenic plants harboring the *p35S::AFB3* construct. We selected two independent lines that presented a 4-fold *AFB3* overexpression level (Supplementary Figure S2). These lines did not show any evident root developmental phenotypic difference when compared to *Arabidopsis* Col-0 (wild type, Col-0 WT) when grown in MS media, however, a strong salt resistance phenotype was observed (Figure 2B). To further characterize this observation, Col-0 WT and *p35S::AFB3* were grown for 3 days in MS plates and then transferred to MS plates (control) or MS plates supplemented with 100 or 150 mM NaCl. Two and five days after NaCl treatment we analyzed changes in root growth and lateral root primordia density (Figure 2A and Supplementary Figure S3). Remarkably, despite the discreet *AFB3* overexpression levels, these lines showed a strong phenotype in terms of salt stress tolerance observed as reduced cotyledon chlorosis (Figure 2B). In Col-0 WT, we observed that primary root growth was strongly inhibited in a concentration-dependent manner (Figure 2C), while lateral root primordia density was maintained constant at 100 mM NaCl and reduced drastically at 150 mM NaCl (Figure 2D). While no changes were observed in control conditions, primary root growth in AFB3 over-expressor lines was less inhibited in response to 100 mM NaCl compared to Col-0 WT under the same treatment (Figure 2C). However, the stronger phenotype was in terms of lateral root primordia density (Figure 2D). When compared to Col-0 WT in control conditions, *p35S::AFB3* over-expression lines showed a marked resistance to salt treatment when compared to Col-0 WT in lateral root primordia density at both 100 and 150 mM of NaCl (Figure 2D). Moreover, at 150 mM the difference with Col-0 WT is even more striking. While Col-0 WT lateral root primordia density is severely reduced at 150 mM, in *p35S:AFB3* lines there is no significant change compared to Col-0 WT control treatments.

Considering the strong phenotype observed in the over-expressor lines, we also evaluated the AFB3 mutant line *afb3-1* [22,26]. In the control condition, this line was affected in root growth and lateral root primordia formation, with a significant reduction of approximately 20% compared to WS (Wassileskaja, wild type) in both parameters (Figure 2E–G). Interestingly, the *afb3-1* mutant was oversensitive at 150 mM NaCl in terms of lateral root primordia density when compared to WS under the same treatment (Figure 2G). This is consistent with the resistant phenotype observed in the over-expressor lines. Altogether, these results suggest a positive role of AFB3 during salt stress response in roots. 

**Figure 3:**
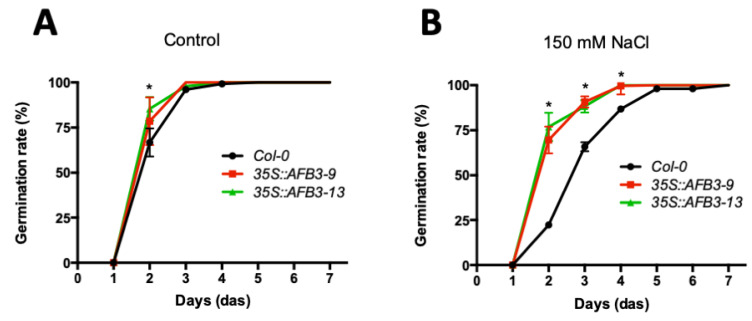
*AFB3* over-expression increases germination rates under control and salt stress conditions. Germination rates of Col-0 WT and *p35S::AFB3* lines were determined on 0.5× MS medium (**A**) and 0.5× MS medium containing 150 mM NaCl (**B**). One-way ANOVA and Bonferroni a posteriori tests were performed. *: *p* < 0.05. Error bars represent the standard error of the mean. Each experiment was performed three times with at least 100 seeds per treatment.

To evaluate whether the salt stress resistance was restricted to root architecture, we evaluated the seed germination rate over a seven-day period under 150 mM NaCl, which is considered an inhibitory salt concentration condition [27]. While no clear phenotype was observed in the *afb3-1* mutant line (Supplementary Figure S4), a difference in the germination rate was observed between both AFB3 over-expressor lines and Col-0 WT at two DAS in control conditions (Figure 3A). While Col-0 WT only reached 60% germination at this point, the *p35S::AFB3* lines showed over 75% germination. This phenotype was even more noticeable under salt stress conditions, where Col-0 WT seedlings exhibited a delay in the germination rate in 150 mM NaCl, reaching only 75% of germinated seeds after three DAS while *p35S::AFB3* lines showed no significant alteration in the germination rate when compared to salt-free media (Figure 3B). Together, these results indicate that over-expressor lines are more resistant to high salt concentrations and are able to germinate normally under this stress condition suggesting a positive role of AFB3 in salt tolerance response.

### 2.3. AFB3-Downstream Signaling Components Are Differentially Regulated in Response to Salt Stress and in AFB3 Over-Expression Lines

**Figure 4:**
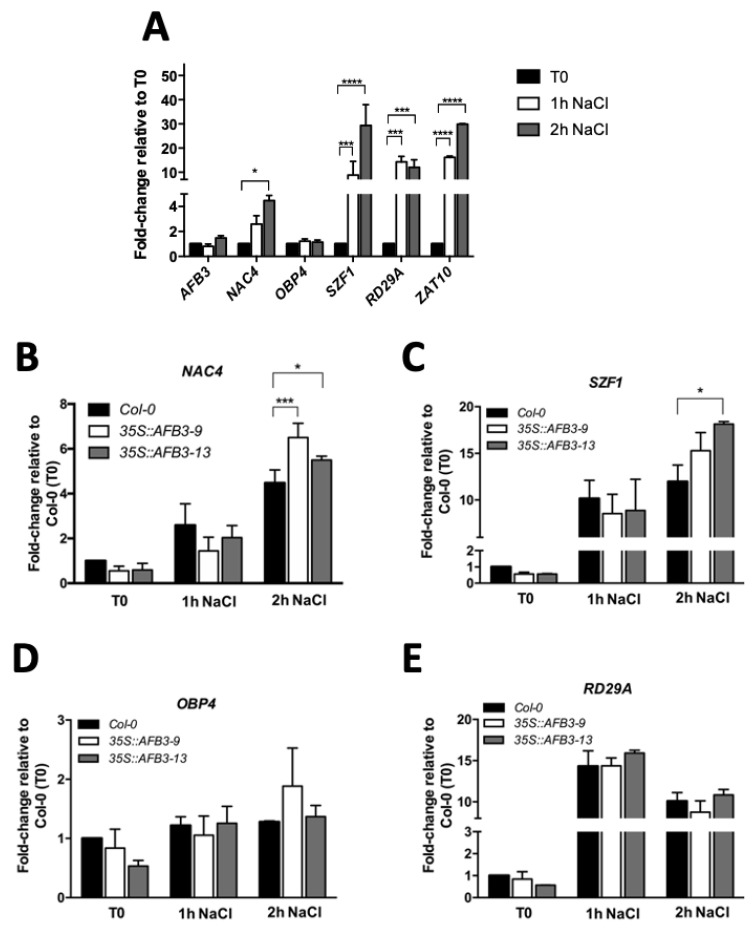
Putative AFB3-downstream signaling components *NAC4* and *SZF1*, are induced under salt stress conditions and differentially regulated in *Arabidopsis thaliana AFB3* over-expressor lines. 7-day-old Col-0 WT and *p35S::AFB3* lines seedlings were transferred onto 0.5× MS medium supplemented with 150 mM of NaCl. At the time of transfer (T0), 1 h and 2 h after NaCl treatment, whole roots were collected. Transcript levels were analyzed by qRT-PCR using *Clathrin adaptor complex subunit* (AT5G46630) as house-keeping gene. (**A**) Col-0 WT, Fold-change was set for T0. (**B**–**E**) Transcript levels from *NAC4* (**B**), *SZF1* (**C**), *OBP4* (**D**), and *RD29A* (**E**) in Col-0 WT and *p35S::AFB3* lines. Fold-change was set for T0 (Col-0 WT). Two-way ANOVA and Bonferroni a posteriori tests were performed. *: *p* < 0.05; ***: *p* = 0.001; ****: *p* < 0.001. Error bars represent the standard error of the mean. Each experiment was performed three times with at least 25 seedlings in each replicate.

Using nitrate as a signaling molecule and a systems biology approach, a regulatory network downstream of AFB3 has been previously proposed [23]. The regulatory network suggested a key role of the transcription factors *NAC4* (no apical meristem (NAM)/*Arabidopsis* transcription activation factor (ATAF)/cup-shaped cotyledon (CUC)), *OBF BINDING PROTEIN 4* (*OBP4*) and *SALT-INDUCIBLE ZING FINGER PROTEIN 1* (*SZF1*). A linear signaling cascade, where OBP4 acts downstream of NAC4, which acts downstream of AFB3 [23]. From these three genes, only *SZF1* was previously described in salt stress responses [28]. To evaluate if *NAC4* and *OBP4* were also participating in salt stress responses, we quantified *NAC4* and *OBP4* transcript levels in response to salt stress (150 mM NaCl) for 1 or 2 h and compared them to *SZF1* together with the previously characterized salt stress markers *RESPONSIVE TO DESICCATION 29A* (*RD29A*) and *SALT TOLERANCE ZINC FINGER* (*STZ/ZAT10*) [29,30]. As expected, there was a marked induction of at least 10 to 15-fold in both stress markers in response to salt stress after 1 and 2 h treatments (Figure 4A). Remarkably, *NAC4* was up-regulated 4–5 fold at 2 h after salt stress treatment while *OBP4* did not show any response suggesting that is not part of the signaling response. Moreover, *SZF1* was highly up-regulated in response to salt stress at both 1 and 2 h of salt stress treatment (Figure 4A). This is in accordance with Sun et al. [28] and the up-regulation is comparable with the response level to salt observed in markers *RD29A* and *STZ/ZAT10*. These results might indicate a common response down-stream of *AFB3* via *NAC4* and possibly *SZF1*.

Since *NAC4* and *SZF1* transcripts are regulated in response to salt stress, we further evaluated whether AFB3 over-expression could lead to the modulation of *NAC4* and *SZF1* transcript levels, either directly or indirectly. Thus, *Arabidopsis* Col-0 WT and *p35S::AFB3* seedlings were grown in MS plates for seven days and then transferred to MS plates with and without 150 mM NaCl and harvested after 1 or 2 h salt treatment. At the selected timepoints root tissue was collected and then qRT-PCR was performed to evaluate *NAC4*, *SZF1*, *OBP4,* and *RD29A* transcript levels. As shown in Figure 4, *NAC4* showed induction at 2 h salt treatment in Col-0 WT and both over-expressor lines. Remarkably, there is also a stronger up-regulation response in the *p35S::AFB3* when compared to Col-0 WT at 2 h NaCl treatments (Figure 4B). A similar situation was observed for *SZF1* (Figure 4C). However, *OBP4* showed no significant difference in either control or salt conditions (Figure 4D). The same was observed for *p35S::AFB3* lines, indicating that *OBP4* is not participating in the AFB3-dependent salt stress responses. Moreover, unlike the differential response observed with *SZF1* and *NAC4*, *RD29A* did not show any differential expression between Col-0 WT and *p35S::AFB3* lines after 1 and 2 h of NaCl treatment (Figure 4E). These results suggest that both *NAC4* and *SZF1* might be participating in the AFB3-dependent response to salt stress. 

### 2.4. nac4 and szf1 Loss of Function Mutants Shows Altered Salt Stress Responses

**Figure 5:**
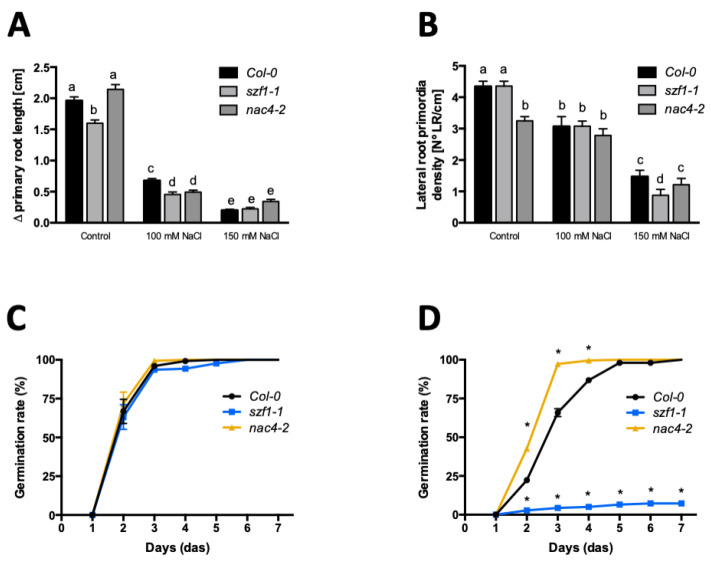
*nac4-2 and szf1-1* mutant lines show an altered response to salt stress in *Arabidopsis thaliana*. (**A**,**B**) 3-day-old Col-0 WT, *nac4-2,* and *szf1-1* lines were transferred to MS medium supplemented with 0, 100, or 150 mM of NaCl for five days. Primary root growth under NaCl treatment (**A**) and lateral root primordia density (**B**) were quantified. (**C**,**D**) Germination rates of Col-0 WT, nac4-1, and szf1-1 plants were determined on MS medium (**C**) and the same medium supplemented with 150 mM of NaCl (**D**). ANOVA and Bonferroni a posteriori tests were performed. *: *p* < 0.05. a, b, c, d represents statistically significant differences with *p* < 0.05. Error bars represent the standard error of the mean. Each experiment was performed in triplicates with at least 12 seedlings (**A**,**B**) or 100 seeds (**C**,**D**) per treatment.

To characterize whether *NAC4* and *SZF1* have a role in salt stress responses we evaluated *nac4* and *szf1* loss of function mutant lines (Figure 5). Col-0 WT, *nac4-2*, and *szf1-1* lines were germinated in MS plates for 3 days. At 3 DAS, seedlings were transferred to MS plates supplemented with or without 100 and 150 mM of NaCl. Plants were grown for 5 days under salt stress treatment. As shown in Figure 5, both *nac4* and *szf1-1* mutant lines were affected in their root architecture. While *szf1-1* had shorter roots, *nac4-2* lines showed a reduced lateral root primordia density (Figure 5A,B). Remarkably, both *szf1-1 and nac4-2* mutant lines showed a mild but significant decrease in primary root growth compared to Col-0 WT in response to 100 mM NaCl treatment (Figure 5A), indicating an impaired sensitivity to salt in terms of primary root length in these mutants. Moreover, LR development was more affected in *szf1-1* in response to 150 mM NaCl, also suggesting an oversensitivity to this stress (Figure 5B).

Constitutive overexpression of *AFB3* showed a marked impact on germination under salt stress conditions (Figure 3). In order to evaluate whether *SZF1* and *NAC4* have a role modulating seed germination under salt stress conditions, *szf1-1* and *nac4-2* mutant lines were evaluated. The germination rate under MS media (control) or 150 mM of NaCl was evaluated for seven days. As shown in Figure 5, no differences were observed in any of the mutant lines compared to Col-0 WT in control conditions, reaching near 100% germination between two- and three-day after sowing (Figure 5C). However, a strong decrease in the germination rate was observed for *szf1-1* in 150 mM of NaCl, reaching only 10% of germinated seeds after seven days after sowing (Figure 5D). Unexpectedly, an opposite phenotype was observed for *nac4-2*, was a salt-resistant phenotype was observed at 3 DAS with near a 100% germination while Col-0 WT only reached 60% (Figure 5D). The root architecture and germination results suggest that the main role of *NAC4* and *SZF1* in salt stress responses is not predominantly *AFB3*-dependent and possibly they are participating in other signaling pathways.

## 3. Discussion

Several studies have attributed the central role of auxin perception to TIR1 and AFB2, which act redundantly and show higher affinity for most auxins [16,21,31,32]. While *TIR1* is up-regulated in response to salt stress [16], at the protein level TIR1 and AFB2 are down-regulated in response to salt, with TIR1 being more sensitive [20]. Interestingly, no significant changes were observed by qRT-PCR in *AFB3* transcript accumulation or GUS expression in *pAFB3::GUS* lines after salt stress (Supplementary Figure S1). Moreover, in contrast with the down-regulation reported for TIR1 and AFB2 GUS protein fusion lines [20], *pAFB3::AFB3-GUS* lines revealed a tissue-specific protein accumulation in response to salt (Figure 1). This tissue-specific modulation strongly suggests a post-transcriptional regulation of the protein abundance. Moreover, this tissue-specific regulation of AFB3 in roots strongly suggests the key role of this auxin receptor in modulating root meristematic activity upon salt stress. The differences in salt stress response between auxin receptors are further supported by the analysis of mutant lines. It has been shown that double mutants *tir1/afb1*, *tir1/afb2,* and *tir1/afb3* have differential responses to salt and oxidative stresses, with *tir1/afb2* being more resistant than the other mutant combinations [17]. In this report, we showed that *afb3* single mutants are hypersensitive to salt stress in terms of lateral root density, suggesting that each receptor also has developmental-specific roles [15,22,25]. Moreover, a higher order of complexity in this auxin perception has been attributed, since some studies have concluded that there are different affinities of these receptors for different auxin molecules and, at the same time, there are different specificities for AUX/IAA and ARF proteins that act downstream of auxin receptors [25,33,34]. All these differences and contrasting responses observed for *TIR1* and AFBs reinforce the idea that auxins can exert differential responses depending on the receptors which might work in a highly complex regulatory network depending on specific environmental and developmental cues. 

To further understand how auxin perception may work in the achievement of tolerance in a salt stress context, we made over-expressor lines of the AFB3 auxin receptor taking into consideration its opposite phenotype observed between *afb3* mutant (Figure 2) when compared to *tir1/afb2* [17]. The over-expression of *AFB3* led to salt resistance in terms of root growth and particularly lateral root density when compared to Col-0 WT seedlings (Figure 2 and Supplementary Figure S4). 

A previous report has shown that salt has a concentration- and exposure-dependent effect in root development [4]. According to this, an over-expression of AFB3 in roots could allow a suitable auxin perception even when the levels of this hormone are lower due to high salt concentrations. *AFB3* overexpression also had a strong impact in seed germination, which is severely affected under salt stress conditions. While *Arabidopsis* seeds germinate between two to three days post-sowing in optimal conditions, under salt stress this is delayed by two days [35]. Surprisingly, *p35S::AFB3* lines showed a normal germination rate under severe salt stress conditions (Figure 3). Although auxin by itself may not be determinant to induce seed germination under salt stress, it has been previously shown that the interaction between auxin and gibberellin may play an important role in several stages of germination and post-germination [36,37]. Thus, the activation of response elements that are responsible for the development of lateral roots under these stress conditions could be stimulated. Moreover, auxin can influence seed germination in the presence of ABA [10]. This may suggest that higher auxin sensitivity mediated by the over-expression of *AFB3* receptor might stimulate hormonal changes in order to induce seed germination in stress conditions.

Although AFB3 transcript was not induced under salt stress in either 1 h or 2 h after salt treatment, *NAC4* and *SZF1*, two putative downstream components described by Vidal et al. [23] were up-regulated in this context (Figure 4). In *p35S::AFB3* lines, both *NAC4* and *SZF1* were up-regulated when compared to Col-0 WT after 2 h salt stress treatment (Figure 4). This suggests that these transcription factors could be downstream components of *AFB3* and that they could have a role in tolerance to salt stress in *p35S::AFB3* lines in terms of modulation of lateral root development. 

As described before, *NAC4* is part of the plant-specific NAC family involved in several developmental processes [38]. In addition, it has been previously described that several members of this family are in auxin signaling, such as *NAC1* and *NAC2* [39,40,41]. In terms of root modulation, it is shown that *nac4-2* seedlings had a miss-regulation on formation of lateral roots in control conditions that were lost under salt stress conditions (Figure 5), suggesting that *NAC4* has a role in the first stages of lateral root development. This is in accordance with a previous study related to *NAC4* in *Caragana intermedia* (*CiNAC4*), a member of Fabaceae, where the over-expression of *CiNAC4* led to an increase in lateral root number but not in primary root growth in long-term salt stress conditions [42]. Remarkably, *nac4-2* mutants showed a resistant phenotype in terms of germination rate under salt stress in this study (Figure 5C,D). Several studies in salt-stress context have attributed a negative role of NAC proteins during seed germination, possibly by the down-regulation of gibberellins [38,43,44].

*SZF1* is a member of the Cys3/Hys (CCCH) Zinc-finger proteins described in *Arabidopsis* [28]. This family has been widely characterized as growth and stress-response components [28,45,46]. Additionally, *SZF1* has been described as a putative auxin-dependent [23] and salt-induced component of theses response pathways [28]. We show here that in AFB3 over-expressor lines there is a significant up-regulation of *SZF1* when compared to Col-0 WT upon salt stress treatment (Figure 4). As expected *szf1-1* mutant line exhibited a marked sensitivity to salt stress, which is in agreement with a previous study carried out by Sun et al. [28]. This suggests that salt-induction of *SZF1* is partially *AFB3*-dependent and it may have a role in the modulation of salt-stress early response in *Arabidopsis*.

It has been described in several zinc-finger family members that their role in the acquisition of tolerance could be related to the modulation of the ABA-responsive genes [47]. This is further highlighted in previous studies where over-expression of zinc-finger proteins such as *SZF1* or *ZFP1* led to a decrease in salt-responsive gene expression and modulation of salt stress tolerance in *Arabidopsis* [28,48]. According to this, it has been proposed that this protein would be negatively regulated by ABA, in the same way as *STZ/ZAT10*, another transcription factor inducible by salt and drought [29]. Furthermore, *SZF1* could act as a repressor of ABA-responsive genes in early stages of salt-stress. Both could provide resistance to salt-stress by modulating the excitatory stress response generated by ABA, enhancing the induction of early response genes responsible for ionic and osmotic adjustment as the activation of the salt-overly-sensitive pathway [8]. All together, these data suggest that in response to salt stress there is an accumulation of AFB3, which contributes to salt stress resistance. This is, at least in part, due to the direct or indirect regulation of *NAC4* and *SZF1*.

## 4. Materials and Methods

### 4.1. Plant Material and Growth Conditions

*Arabidopsis thaliana* wild-type ecotype Columbia-0 (Col-0 WT) and Wassilewskija (WS) were used in this work and they are indicated in each section accordingly. *Arabidopsis* knockout mutants *szf1-1* (SALK_141550), *nac4-2* (SALK_006735), and *afb3-1* (SALK_068787) were obtained from the Salk Institute Genomic Analysis Laboratory T-DNA insertion mutant seed collection (donated to the *Arabidopsis* Biological Resource Center). *pAFB3::GUS* (CS69678) and *pAFB3::AFB3-GUS/afb3-4* (CS69682) lines were obtained from the *Arabidopsis* Biological Resource Center [25]. Primers used for genotyping each T-DNA line are listed in Supplementary Table S1. *Arabidopsis thaliana* seeds were incubated in water at 4 °C over-night. Seeds were then sterilized in Eppendorf tubes using a 2.5% Sodium hypochlorite, 0.1% Triton X-100 solution under constant mixing for 7 min. Then seeds were washed with sterile water at least three times for 3 min each time under constant mixing. After sterilization, seeds were sown on squared Petri dishes containing 0.5× Murashige and Skoog (MS) media supplemented with 0.05% MES, 1% sucrose, 0.8% agar. pH was adjusted at 5.8 using potassium hydroxide (KOH) 1 M. For plant growth, growth chambers were used with a long-day photoperiod (16 h light/8 h dark) and 22 °C constant temperature. For salt stress treatments, 100 or 150 mM of sodium chloride (NaCl) were added in previously described 0.5× MS medium.

### 4.2. Generation of Overexpression Lines

For the generation of *AFB3* overexpression lines, the ORFs were amplified from a complementary DNA (cDNA) template with Pfu DNA Polymerase (Promega, Madison, WI, USA) using primers containing the Gateway attB sites and cloned into pDONR221 to generate the *AFB3* ENTRY vector. The *AFB3* ORG in the entry vector was cloned in the binary vector pK7m24GW using the *CaMV p35S* promoter, thus generating *p35S::AFB3* lines. Overexpression lines were generated by floral dip method [49]. For the selection of *p35S::AFB3* over-expression lines, seeds of 22 independent lines were sown in MS medium supplemented with 1% sucrose, 0.8% agar with 25 µg/mL of Kanamycin. *p35S::AFB3* seedlings from resistant lines were grown in substrate and propagated for further experiments. Later, AFB3 gene expression levels of the previously selected lines were performed by using quantitative polymerase chain reaction.

### 4.3. Salt Treatment

For short-term salt treatments, *Arabidopsis thaliana* seeds were sterilized as described in Methods Section 1 and sown on 0.5× MS Medium pH 5.8. Seedlings were grown for 7 days in a growth chamber. Seven-day-old seedlings were transferred onto 0.5× MS medium with 0 or 150 mM NaCl for 1 or 2 h. For longer treatments, *Arabidopsis thaliana* seeds were grown for 3 days in growth chambers and then transferred to MS media supplemented with 0, 100, or 150 mM NaCl for 2, 5, or 10 days.

### 4.4. Phenotypic Analysis

Primary root growth was analyzed from seedling images recorded at given timepoints with the Perfection V700 Photo scanner (EPSON, Nagano, Japan). Roots were measured using ImageJ Software (National Institutes of Health, Bethesda, MD, USA; http://rsb.info.nih.gov/ij). To score lateral root primordia density, root clearing was performed according to Malamy and Benfey [50] with minor modifications. Briefly, seedlings were incubated in a 70% Ethanol solution overnight. Then a solution containing 20% Methanol and 4% HCl was added and seedlings were incubated for 40 min at 62 °C. This solution was replaced with a 60% Ethanol, 7% NaOH solution and incubated for 20 min at room temperature. Roots were re-hydrate in a succession of 40%, 20%, and 10% Ethanol solutions for 20 min each. Finally, an equal volume of 50% glycerol was added which made a 5% ethanol, 25% glycerol solution, and incubated for 2 h. Cleared seedlings were mounted in 25.4 × 76.2 mm microscope slides with 50% glycerol and covered with 24 × 50 mm cover glass [50]. The number of lateral roots was analyzed using a differential interference contrast microscope Zeiss (Berlin, Germany). Total lateral root density was calculated as the ratio between the total number of the lateral roots and the primary root length of each plant. 

To evaluate germination potential under salt stress conditions, seeds were sown on MS medium with 0 or 150 mM of NaCl. Seed germination was scored daily for 7 days. Germination of the seed was established by root protrusion.

### 4.5. Histochemical Analysis

To detect β-glucuronidase (GUS) activity, 7-day-old seedlings were incubated in reaction buffer containing 0.1 M sodium phosphate buffer (pH 7), 1 mM ferricyanide, 1 mM ferrocyanide, 0.1% Triton X-100 and 1 mg mL^−1^ X-Gluc for 30 min up to 24 h in dark at 37 °C. Afterwards, chlorophyll was removed by incubation in 70% ethanol and seedlings were cleared as previously described [50]. GUS staining in roots was imaged by differential interference contrast microscopy Olympus BX51 (Tokyo, Japan).

### 4.6. DNA Purification

Genomic DNA was extracted from leaf tissue of 4-6-week-old plants. The purification of genomic DNA was performed according to a modified protocol, originally performed by Richards et al. [51]. Briefly, tissue samples were pulverized and 200 µL of High-salt TE extraction Buffer (200 mM Tris-HCl pH 8.0, 25 mM EDTA pH 8.0, 250 mM NaCl and 0.5% p/v SDS) was added. Samples were centrifugated at 12,000 RPM for 5 min. Supernatant was transferred onto a new microcentrifuge tube with 200 µL of Isopropanol to precipitate the DNA present in the samples. Then samples were mixed for 15 min and centrifugated at 12,000 RPM for 5 min. Supernatant was discarded and pellet was washed with 200 µL of 70% ethanol. To precipitate the DNA, samples were centrifugated at 12,000 RPM for 5 min and supernatant was discarded. Finally, DNA samples were dried and re-suspended in 100 µL of DNAse/RNAse free water [51].

### 4.7. RNA Extraction and cDNA Synthesis

Roots from 7-day-old or 10-day-old *Arabidopsis thaliana* seedlings were collected and frozen in liquid nitrogen. RNA purification was performed using the Purelink RNA Mini Kit (Thermo Fisher Scientific, Waltham, MA, USA) following manufacturer instructions. Then RNA samples were treated with DNase I Amplification Grade kit (Thermo Fisher Scientific, Waltham, MA, USA) to finalize RNA purification. RNA samples were treated with DNase I for 15 min at room temperature. To inactivate the enzyme, 1 μL of 25 mM EDTA was added and samples were incubated at 65 °C for 10 min. RNA samples were quantified and purity checked using the Nanodrop 2000 spectrophotometry system (Thermo Fisher Scientific, Waltham, MA, USA). 

cDNA synthesis was performed by the incubation of 500 ng of each total RNA sample with 1 µL oligo dT (0.5 µg/µL) at 70 °C for 5 min to amplify only the mRNAs in the sample. Samples were retrotranscribed to DNA using “Improm II Reverse Transcriptase” include manufacturer instructions.

### 4.8. Quantitative Polymerase Chain Reaction (qPCR)

qPCR of cDNAs was performed using Brilliant III Ultra-Fast SYBR Green qPCR kit (Agilent Technologies, Santa Clara, CA, USA) following manufacturer instructions. cDNA of each gene was amplificated using primers listed in Supplementary Table S1. The reaction was performed in StepOnePlus^™^ Real-Time PCR System (Applied Biosystems, Waltham, MA, USA) in 0.1 mL MicroAmp^®^ Fast 8-Tube Strip (Applied Biosystems, Waltham, MA, USA). The program used for the amplification went as follows: 95 °C for 3 min, 95 °C for 5 s and 60 °C for 10 s during 40 cycles and a final elongation for 15 s at 95 °C. Additionally, a melting curve was performed from 65 °C to 90 °C rising 0.3 °C each phase to confirm the amplification of only one transcript on each gene.

Data were normalized using *clathrin adaptor complex subunit* (AT5G46630) as housekeeping gene [52]. Finally, data were analyzed using LinReg program [53]. 

### 4.9. Statistical Analysis

All experiments were performed at least three times. For primary root growth, lateral root density, and qRT-PCR analysis, two-way ANOVA and Bonferroni a posteriori test were performed. Statistically significant *p* values were set to < 0.05. For germination rate analysis, One-way ANOVA and Bonferroni a posteriori test were performed. Statistically significant *p* values were set to < 0.05.

## Supplementary Materials

The following are available online at https://www.mdpi.com/1422-0067/21/24/9528/s1.
