# Supplementary material for: Overexpression of the Auxin Receptor AFB3 in Arabidopsis Results in Salt Stress Resistance and the Modulation of NAC4 and SZF1
Source: Int J Mol Sci. 2020 Dec 15;21(24):9528. doi: 10.3390/ijms21249528 (PMC7765236; doi:10.3390/ijms21249528)
Supplement: Supplementary file 1 [file ijms-21-09528-s001.pdf]

Supplementary Materials

Overexpression of the Auxin Receptor *AFB3* in *Arabidopsis* Results in Salt Stress Resistance and the Modulation of *NAC4* and *SZF1*

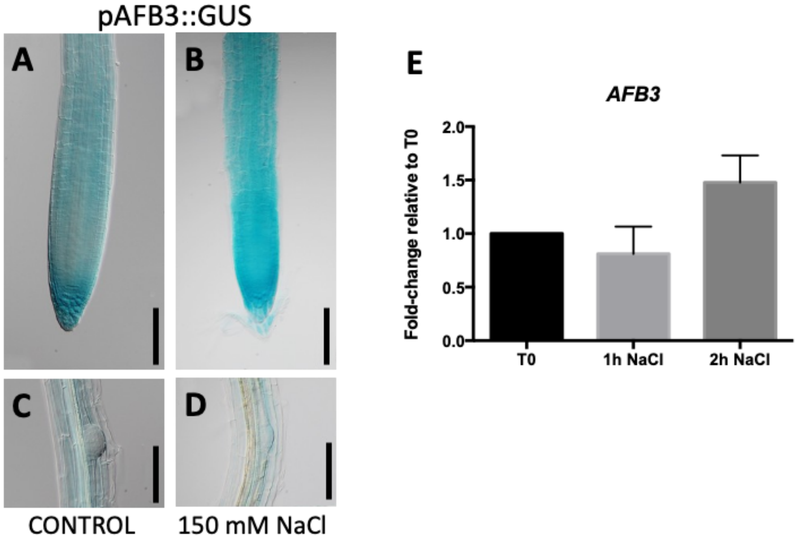

Figure S1. *AFB3* auxin receptor is not induced in roots at the transcriptional level under salt stress response. 3-days-old *pAFB3::GUS* (**A**,**B**,**C**,**D**) lines were transferred onto MS medium supplemented with 0 (**A**,**C**) or 150 mM of NaCl (**B**,**D**) for five days. At day 8 (das), seedlings were subjected to β-Glucuronidase (GUS) staining. A representative picture of each treatment is shown. (**A**,**B**) Meristematic/Elongation zone, (**C**,**D**) Lateral root. Bar = 0.2 mm. (**E**) 7-days-old Col-0 WT seedlings were transferred onto 0.5× MS medium supplemented with 150 mM of NaCl. At the time of transfer (T0), 1 h and 2 h after NaCl treatment, whole roots were collected. *AFB3* transcript levels were analyzed by qRT-PCR using *Clathrin adaptor complex* as house-keeping gene. Fold-change was set for Col-0 WT (T0). Two-way ANOVA and Bonferroni a posteriori tests were performed and no significant differences were observed. Error bars represent the SEM. Each experiment was performed in triplicate with 25 seedlings each replicate.

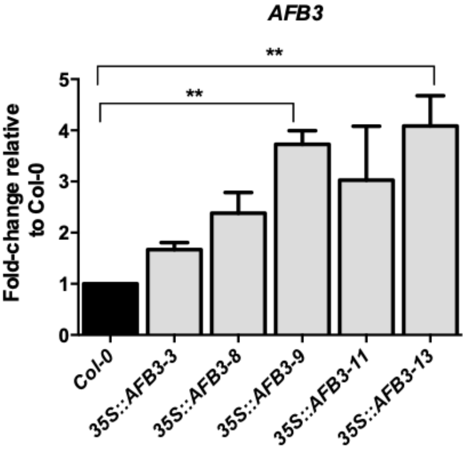

Figure S2. *p35S::AFB3* independent lines express higher levels of *AFB3* transcripts in roots. Roots from 10-days-old seedlings of each over-expression line and wild-type ecotype Col-0 WT were collected and qRT-PCR were performed. *AFB3* mRNA levels were normalized using *Clathrin adaptor complex* as a house-keeping gene. Fold-change was set using *AFB3* mRNA levels from Col-0 WT. One-way ANOVA and Bonferroni a posteriori tests were performed. **: $p < 0.005$; Error bars represent the Standard Error of the Mean (SEM). Each experiment was performed three times with $n = 30$.

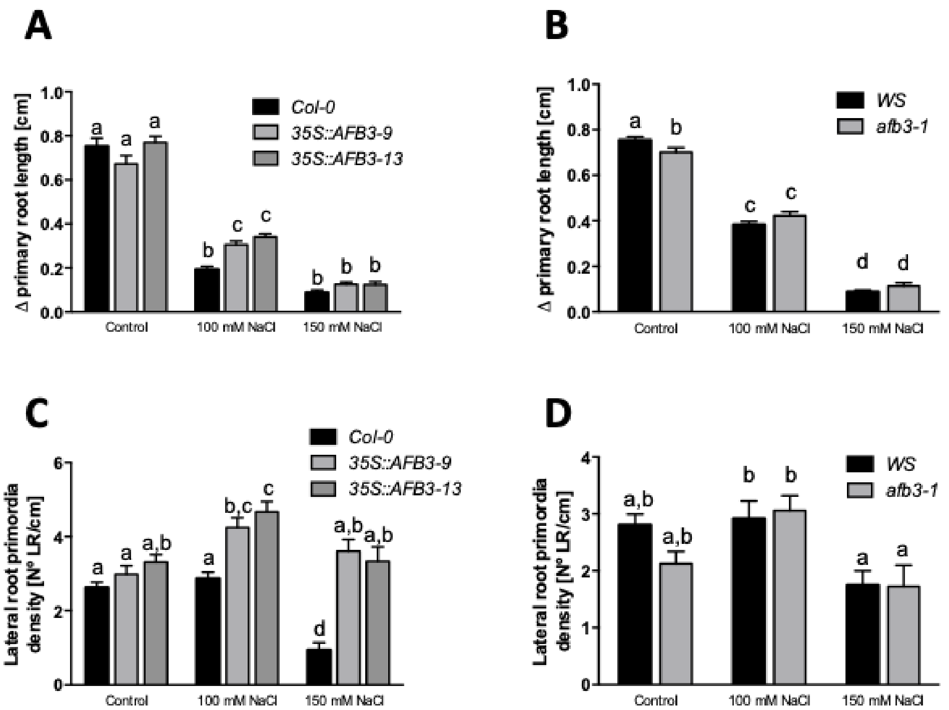

Figure S3. *AFB3* over-expression enhances salt stress tolerance in roots. 3-days-old Col-0 WT, *p35S::AFB3*, WS and *afb3-1* were transferred onto MS medium supplemented with 0, 100 or 150 mM of NaCl for two days. (**A**,**B**) Primary root growth after transfer to NaCl containing media and (**C**,**D**) lateral root primordia density was quantified. Two-way ANOVA and Bonferroni a posteriori tests were performed. a, b, c, d represents statistically significant differences with $p < 0.05$. Error bars represent the SEM. Each experiment was performed at least three independent times, with 12 seedlings each replicate.

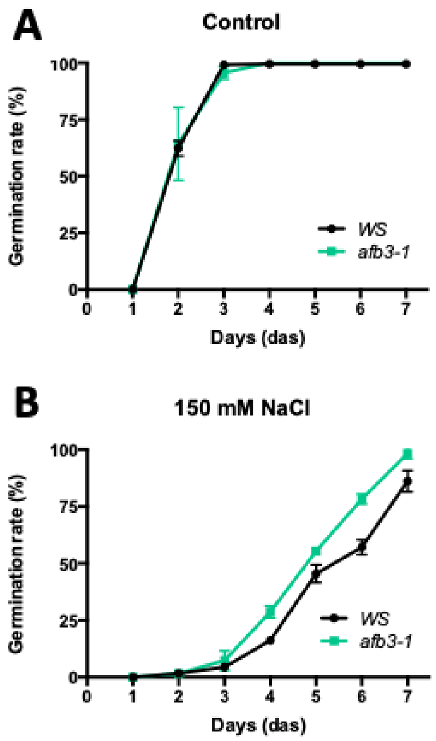

Figure S4. Impaired *AFB3* expression in *afb3-1* mutant does not affects germination rates under salt stress conditions. Germination rates of Col-0 WT and *afb3-1* mutant line were determined on 0.5× MS medium (**A**) and 0.5x MS medium containing 150 mM NaCl (**B**). One-way ANOVA and Bonferroni a

posteriori tests were performed and no significant difference was observed. Each experiment was performed three times with at least 100 seeds per treatment.

Table S1. DNA Sequences of primers for qRT-PCR and genotyping.

**qRT-PCR Primers**		
**Primer Name**	**Gene**	**Primer Sequence (5′–3′)**
AtAFB3 fw	*At1G12820*	aggaagctggagataagggacagt
AtAFB3 rv	*At1G12820*	aagggatcgcattgtttcgt
AtNAC4 fw	*At5G07680*	ttcccttagctccatccaaccaga
AtNAC4 rv	*At5G07680*	cttgcgtaagaaccggattgga
AtSZF1 fw	*At3G55980*	cgcagctagagactacagggac
AtSZF1 rv	*At3G55980*	agcttctctcagacaccacagtac
AtCLAT fw	*At4G24550*	aatacgcgctgagttccctt
AtCLAT rv	*At4G24550*	agcaccgggttctaactc
AtOBP4 fw	*At5G60850*	gaacggctcaggttgagttt
AtOBP4 rv	*At5G60850*	tgcatgatcaacggtactgg
AtRD29A fw	*At5G52310*	atcacttggctccactgttgttc
AtRD29A rv	*At5G52310*	acaaaacacacataaacatccaaagtg
AtZAT10 fw	*At1G27730*	gagtcgagcactggacaaagg
AtZAT10 rv	*At1G27730*	gagcgagaagcatgaggcaa
**Genotyping Primers**		
**Primer Name**	**Gene**	**Primer Sequence (5′–3′)**
nac4-2 fw	*At5G07680*	tgaggactaaccgagcaactc
nac4-2 rv	*At5G07680*	aacccaccacaatgcattaac
szf1-1 fw	*At3G55980*	agaagagtcagcacaagagcg
szf1-1 rv	*At3G55980*	ttccagtggaaacgatgaaag
afb3-1 fw	*At1G12820*	tcatgttgcttacaaattgcg
afb3-1 rv	*At1G12820*	tctgcaaacagatgacaaacgaaacg
